# Criterion-Related Validity and Reliability of a Measurement Tool for Medical Doctors’ Work-Related Quality of Life in Japan

**DOI:** 10.3390/jmahp13040057

**Published:** 2025-11-14

**Authors:** Miyuki Ezura, Katsuhiko Sawada, Yusuke Takushima, Lida Teng, Ataru Igarashi

**Affiliations:** 1 Department of Corporate Planning, Otsuka Holdings Co., Ltd., Tokyo 108-8042, Japan; 2 Department of Health Technology Assessment Program, Graduate School of Health Management, Keio University, Tokyo 160-8582, Japan; 3 Clinical Development Department, Otsuka Medical Devices Co., Ltd., Tokyo 108-0075, Japan; sawada.katsuhiko@otsuka.jp; 4 Public and Government Affairs Department, Taiho Pharmaceutical Co., Ltd., Tokyo 101-8444, Japan; y-takushima@taiho.co.jp; 5 Department of Health Economics and Outcomes Research, Graduate School of Pharmaceutical Sciences, The University of Tokyo, Tokyo 113-0033, Japan; lidateng@gmail.com (L.T.);

**Keywords:** healthcare, quality of life, questionnaire, validity, reliability, medical doctor

## Abstract

**Objective:** This confirmatory survey aimed to verify the criterion-related validity and reliability of the final version of the Medical Doctors’ Work-Related Quality of Life Questionnaire (WQMD-9), following partial revision of its content. This study also explored the questionnaire’s structure and scoring methods. **Method:** From June to July 2022, the WQMD-9 was administered to 98 MDs selected to match the statistical distribution of MDs in Japan. Criterion-related validity was evaluated using a visual analogue scale (VAS) as the reference standard, and reliability was examined using inter-dimension correlations and Cronbach’s α. **Results:** The correlation coefficient between the VAS score and the simple sum of WQMD-9 dimensions scores was 0.7891, supporting criterion-related validity. Cronbach’s α was 0.87, indicating acceptable reliability. **Conclusions:** The profile-type WQMD-9 consists of nine dimensions—“Workload,” “Working time,” “Collaboration,” “Clinical practice,” “Working conditions,” “Working environment,” “Feelings of fatigue,” “Work-life balance,” and “Career”—with five levels. In the confirmatory survey population, the WQMD-9 demonstrated criterion-related validity and reliability, suggesting that it can be utilized with simple total scoring approach.

## 1. Introduction

The emergence of game-changing healthcare technologies in recent years has upended conventional approaches in medical device and pharmaceutical development [1,2]. These advancements are considered to deliver value not only to patients but also to healthcare providers by significantly influencing clinical practice [3,4]. The Medical Doctors’ Work-Related Quality of Life Questionnaire (WQMD-9) was developed to visualize these challenges among MDs (Figure S1). Unlike resource use metrics such as procedure duration or hospitalization period, WQMD-9 offers a subjective assessment of MDs-centered perspective. To begin with, we performed a systematic review of existing questionnaires that assess work-related quality of life (QOL) in the context of MDs [5].

From the final selection of 81 papers revealed that existing studies assessing MDs’ quality of life (QOL) typically employed a combination of original items and multiple standardized instruments, rather than a single unified questionnaire. For example, a study conducted in Denmark utilized the Maslach Burnout Inventory–Human Services Survey (MBI-HSS) to assess fatigue, the Warr–Cook–Wall Job Satisfaction Scale (WCW-JSS) for career satisfaction, the Perceived Stress Scale (PSS-10) for working environment, and both the Short-Form Health Survey (SF-12) and the World Health Organization Five Well-Being Index (WHO-5) for overall QOL, while work–life balance was evaluated using original items [6]. Although comprehensive assessment using multiple validated instruments is a valid approach, it presents challenges such as increased respondent burden and interpretive complexity when integrating results across different scales. In this context, the development of a single consolidated questionnaire offers significant advantages for both researchers and participants. We therefore decided to develop a new questionnaire, for which we defined the “work-related QOL of medical doctors” as factors related to a medical doctor’s work (such as clinical practice) that subjectively impact their own satisfaction, work, and life, and we constructed a profile-type QOL scale accordingly. In our previous research [7], we developed the WQMD-9 which is an original profile-type scale consisting of nine dimensions and five levels. We followed a standard methodology for the development of a QOL scale, beginning with qualitative interviews of 20 MDs, then validation interviews with 8 MDs, and finally a quantitative survey with 374 MDs to evaluate its validity, inter-dimension correlations, and reliability. According to this survey’s results, the response wording was modified, and a temporal element was added to the beginning of each question to avoid ceiling effects. The WQMD-9 was then finalized after consultation with external experts. Notably, the WQMD-9 is the first tool developed in Japan by our research team to assess the work related QOL for MDs [8].

In this study, to examine the characteristics of the profile-type scale, we aimed to verify the criterion-related validity and reliability of the finalized WQMD-9—which has already been validated for internal consistency, content validity, construct validity, and interpretability [9]—and to conduct an explorative analysis of its construct validity through factor analysis of the questionnaire’s structure (dimensions) and consideration of scoring methods.

## 2. Materials and Methods

A confirmatory survey using the WQMD-9 was conducted with 98 MDs [10] recruited from an MD registry maintained by a research company, according to criteria based on the distribution proportions published by the Ministry of Health, Labour and Welfare in the 2018 National Medical Doctor Statistics [11]: hospital MDs (67%)/private practitioners (33%); internal medicine specialties (51%)/surgical specialties (29%)/others (19%); male (79%)/female (21%); and age groups 29 years and under (9%), 30–39 years (21%), 40–49 years (22%), 50–59 years (22%), 60–69 years (17%), and 70 years and above (10%). Recruitment was conducted via the internet, with enrollment for each stratum closed once the target number was reached. To evaluate criterion-related validity, we added a question on “overall work-related QOL” using a visual analogue scale (VAS) [12]. A prior systematic review [5] found that there was no single questionnaire designed to measure work-related QOL among MDs; thus, the VAS was employed for the purpose of measuring overall QOL. To examine internal consistency, we assessed inter-dimension correlations and calculated Cronbach’s α coefficient. In addition, to reconfirm the questionnaire structure identified in the previously conducted quantitative survey [7], we conducted exploratory factor analysis and examined scoring methods. However, as we do not currently intend to use the two identified factors (dimensions) separately, we also performed exploratory analyses in our survey with 98 MDs. Data analysis was then performed using JMP Version 16 statistical software (JMP Statistical Discovery LLC, Cary, NC, USA).

Following completion of both the confirmatory survey and exploratory analyses, an external expert panel review was conducted to finalize the WQMD-9.

This survey was exempt from ethics committee review, as it focused on instrument development and validation rather than collecting data on individual behaviours or perceptions. Prior to participation and via an online platform, all individuals were presented with a detailed outline of the purpose of the study, the voluntary nature of their involvement, the restricted use of data (i.e., data would be utilized exclusively for scale development), and the complete anonymization of all responses. Informed consent was obtained electronically and documented appropriately.

## 3. Results

The results of the confirmatory survey conducted with 98 MDs between June and July 2022 are presented below. For clarity, we refer to the questionnaire version tested in the initial quantitative survey of 374 MDs during the first stage of scale development as “pre-WQMD-9,” while the revised version evaluated in this confirmatory study is referred to as “WQMD-9.”

### 3.1. Criterion-Related Validity

**Figure 1:**
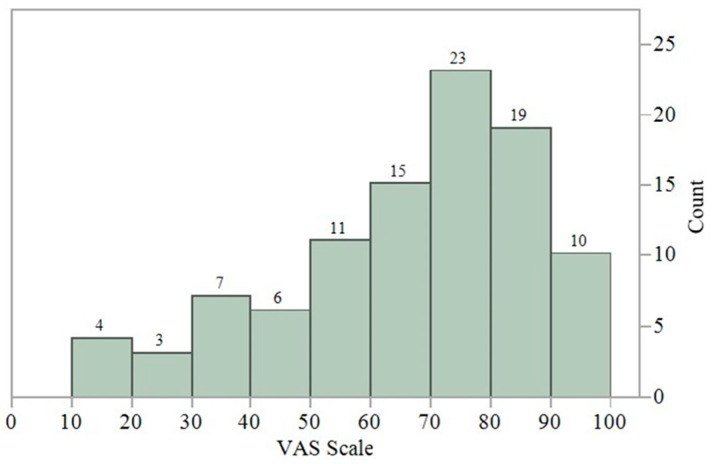
Distribution of responses to the visual analogue scale (Q10).

Figure 1 shows the distribution of responses to the VAS (Q10), which was administered along with the nine-dimension WQMD-9 to examine criterion-related validity. The mean VAS score was 64.7 (median: 70.0), and values ranged from a minimum of 15 to a maximum of 100. The correlation coefficients (Spearman’s rho) between the VAS and the nine constituents’ dimensions of the WQMD-9 were calculated to assess criterion-related validity. Correlations were generally strong to moderate in strength (r = −0.46 to −0.79), although the correlation with Q3 (“Collaboration”) was notably weaker than the others (r = −0.25; see Table 1).

### 3.2. Reliability

Table 1 presents the inter-dimension correlation coefficients, which were calculated to assess the internal consistency of the questionnaire. The strongest correlation was observed between Q5 (“Working conditions”) and Q6 (“Working environment”), with a coefficient of 0.8324. The correlations between Q3 (“Collaboration”) and other dimensions were generally the weakest, with coefficients as low as 0.162. Cronbach’s α coefficient calculated from the responses to dimensions Q1–Q9 was 0.899, indicating the WQMD-9′s good internal consistency.

### 3.3. Resolution of Ceiling Effects

**Figure 2:**
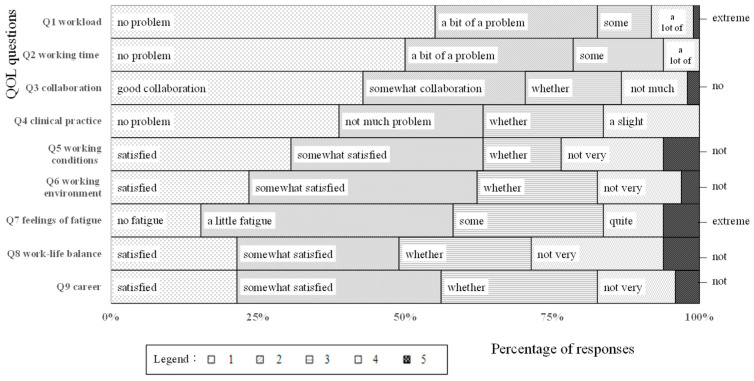
Distribution of responses to dimensions Q1–Q9.

The response wording for Q4 (“Clinical practice”) was modified for the confirmatory survey, as the response distribution exhibited a problematic ceiling effect in the quantitative survey conducted during the first stage of scale development. Figure 2 shows a mosaic plot of the responses to Q1–9. For Q4, the confirmatory survey’s results showed increased responses for “3” and “4,” indicating that the ceiling effect was successfully resolved. The responses to the other question dimensions were generally similar to those of the initial pre-WQMD-9 survey, with no problematic ceiling effects identified.

### 3.4. Exploratory Analysis

#### 3.4.1. Factor Analysis

**Figure 3:**
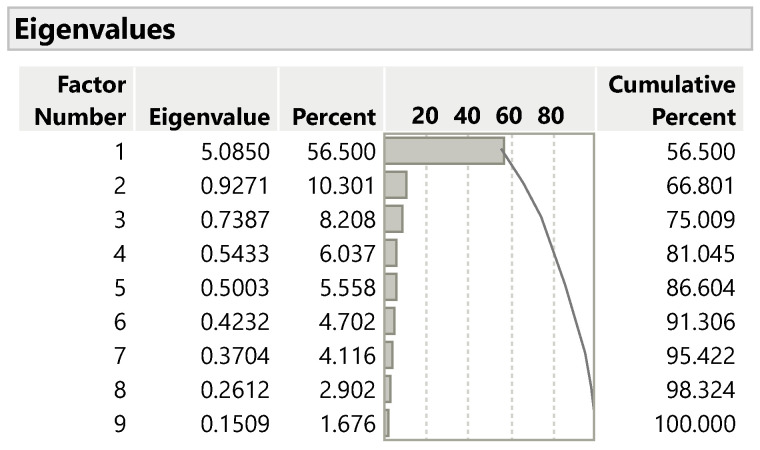
Eigenvalues and percentage contribution of factors.

The eigenvalues, corresponding factor contribution rates, and cumulative plots are shown in Figure 3. The exploratory analysis suggested that one to two factors would be appropriate.

Table 2 shows the factor loadings estimated by the maximum likelihood method when specifying a single-factor solution.

Table 3 shows the factor loadings estimated by the maximum likelihood method with Promax oblique rotation when exploring a two-factor solution. The results suggest that it may be possible to separate dimensions into a factor representing “labor” (Q5 and Q6) and a factor representing “work” (Q1 and Q2), subsequently referred to as Factor A and Factor B, respectively. This analysis showed similarities with the exploratory factor analyses conducted in our previous survey [6]; however, some dimensions overlapped between factors and could not be categorized into one (e.g., Q3, Q4, Q7 or Q8).

#### 3.4.2. Scoring Method Comparison

Candidate scoring methods were compared. Table 4 displays the results of the factor analysis using standardized scores. Responses were calculated as equidistant ordinal scales for this analysis.

Table 5 presents the correlation coefficients between the QOL VAS, simple sum values, and factor scores derived from the one- and two-factor models. Correlations were estimated using listwise deletion. Within the two-factor model, Factor A, which represents dimensions related to “labor”, showed higher correlation with the QOL VAS.

### 3.5. Summary of Results

While VAS is not a standardized reference measure, the criterion-related validity of the WQMD-9 can be considered confirmed with relation to work-related QOL VAS, as provides supportive evidence for the correlation (r = 0.7891) between the simple sum value and VAS score. The results of the scoring method comparison further indicate the suitability of unweighted simple summation without the need for additional item weighting, as demonstrated by the similar correlation coefficients between the VAS and simple sum (r = 0.7891) and between the VAS and the one-factor solution (r = 0.7824).

The results presented confirm the robust psychometric properties of the revised version of the WQMD-9 tested in the confirmatory survey, with no ceiling effects in the response distributions. Furthermore, the scale demonstrated reliability in the study population, who were demographically matched with the broader population of Japanese MDs (the intended users of this questionnaire), as evidenced by Cronbach’s α coefficient of 0.899.

Following expert panel review, the WQMD-9 was finalized as a nine-dimension instrument, with each dimension measured on five levels: “Workload,” “Working hours,” “Collaboration,” “Clinical practice,” “Working conditions,” “Working environment,” “Psychology,” “Work-life balance,” and “Career” ([7] and Figure S1 is in Japanese as originally written).

## 4. Discussion

This study was conducted to confirm the validity of a prior quantitative survey conducted with 374 MDs, while this survey was carried out with 98 MDs to evaluate criterion-related validity and assess the impact of differences in study populations on reliability. While a sample of 98 MDs is not statistically adequate for a rigorous standalone study, it exceeds the minimum threshold of ten times the number of questionnaires dimensions (i.e., 10 × 9 dimensions = 90) [13]. Therefore, as a confirmatory survey supporting a larger-scale survey of 374 MDs, we consider it sufficient. We compared the results with those of a previous quantitative survey [7] because, although the two study populations had similar attributes and distributions, they were not identical.

Regarding internal consistency, the inter-dimension correlation coefficients ranged from 0.162 to 0.832 in the confirmatory survey cohort (WQMD-9), compared with 0.328 to 0.699 in the quantitative survey cohort (pre-WQMD-9). The weakest correlation in the confirmatory survey was between “Collaboration” and “Career” (0.162), similarly to the quantitative survey (0.328). The highest correlation in the quantitative survey was between “Workload” and “Working hours” at 0.699 (0.694 in the confirmatory survey), while in the confirmatory survey, the highest correlation was between “Working environment” and “Working conditions” at 0.832 (0.468 in the quantitative survey). The temporal element added to the questions in the confirmatory survey may have influenced the correlation between “Working environment” and “Working conditions.” However, the correlations of Q4 “Clinical practice” with the other dimensions remained almost identical between the quantitative and confirmatory survey groups, suggesting that changing the response format did not affect reliability. These results suggest that the differences in study populations did not affect the reliability of the WQMD-9. Regarding criterion-related validity, our systematic literature review [5] revealed that no existing tools exclusively measure work-related QOL among MDs. Therefore, we employed a visual analogue scale (VAS) to assess overall work-related QOL. However, while the VAS provides a simple and intuitive means of capturing subjective evaluations, it lacks the psychometric rigour and dimensional specificity, and theoretical grounding necessary for rigorous validation [14,15]. Its use in this context represents a methodological compromise by the absence of more suitable tool, and the resulting findings should be interpreted with considerable caution. In the quantitative survey, the validity of individual dimensions was assessed by examining the relationships between each WQMD-9 dimension and the corresponding original dimensions [7]. However, to develop a more robust and clinically applicable instrument, it will be necessary to evaluate each component separately using appropriate standardized measures. For instance, the WHOQOL [16] may be used to assess overall quality of life, the Maslach Burnout Inventory [17] can be applied as a validated tool for measuring burnout, the Karasek Job Content Questionnaire (JCQ) [18] is applicable for evaluating work environment factors, and the Effort–Reward Imbalance (ERI) Questionnaire [19] is suitable for assessing occupational stress related to perceived effort and reward. Further validation of the WQMD-9 using these established instruments is warranted to ensure its reliability and domain-specific accuracy. While the WQMD-9 still requires further validation, particularly at the dimension level, its creation represents a meaningful first step toward establishing a unified tool for assessing work-related QOL among medical doctors.

Next, we present a discussion of the exploratory factor analysis and scoring procedures, noting that the results are preliminary and should be considered tentative. Based on the results of the exploratory factor analysis performed during the development of the Pre-WQMD-9, we anticipated that it may be possible to separate the questionnaire dimensions into two factors: one factor with high loadings for Q1 “Workload” and Q2 “Working hours” and a second factor with high loadings for Q6 “Working environment”, Q5 “Working conditions”, Q8 “Work-life balance”, and Q9 “Career”. In this survey, the exploratory two-factor analysis showed relatively similar results to that of a survey for pre-WQMD-9, despite some modest differences in the factor loadings of some questionnaires. Considering the limitations of these exploratory analyses, a prospective survey for confirmatory factor analysis will be very important for drawing meaningful conclusions, as well as addressing the observed cross-loadings, if our hypothesis of two factors is to be used for this instrument in the future.

Furthermore, Q3 “Collaboration” showed the least correlation with the VAS and the lowest contribution to the factors in the exploratory factor analysis. We did not exclude Q3 from the WQMD-9 questionnaire due to the nature of these exploratory analyses; however, the content of Q3 may require further scrutiny. The scoring method comparison in this survey suggested that unweighted simple sum scores may be sufficient for practical applications, such as internal comparisons between interventions or timepoints, although more complex weighted scoring methods may have advantages for the external validity of these scores. Future research should focus on accumulating additional data through expanded prospective confirmatory surveys to further refine and validate the WQMD-9.

Finally, the WQMD-9 was developed for Japanese MDs and created in Japanese; as English validation has not been conducted, we address here the issue of generalizability. As with other healthcare workers, the components of MDs’ work-related QOL may vary depending on the healthcare system. In Japan [19], medical fees are the same regardless of age, experience, or skill level, and the system follows a social insurance model with free access. In the United Kingdom [19], most MDs are civil servants under a general practitioner (primary care) system funded through taxation. In the United States [20], apart from Medicare and Medicaid, the system is largely private, with MDs’ compensation determined, to a large extent, at the discretion of the provider. Even among these three countries, the differences in healthcare systems are substantial, and their influence on QOL remains unclear. Nevertheless, if the “definition of a MDs” [21] under the World Medical Association’s Declaration of Genevais consistent across countries, the elements constituting QOL would be expected to remain largely unchanged. However, if environmental differences are found to influence these elements, it may be necessary to consider approaches such as applying score weighting or developing an index-type questionnaire that allows for international comparisons.

## 5. Conclusions

This study demonstrated the criterion-related validity of the WQMD-9, a tool for measuring work-related QOL among MDs, and confirmed its reliability, with no notable differences with the results of a previous quantitative survey. However, the fact that criterion-related validity was examined using a VAS is an acknowledged limitation: future studies should also verify validity using established questionnaires that are suitable for each dimension. The profile-type WQMD-9 was specifically designed for Japanese MDs: by enabling more precise visualization of the benefits for this demographic, which has often been overlooked, it represents a highly meaningful achievement as a new evaluation metric.

## Figures and Tables

**Table 1:** Correlation coefficients between the VAS and the nine constituents’ dimensions.

	Q1 Workload	Q2 Working Time	Q3 Collaboration	Q4 Clinical Practice	Q5 Working Conditions	Q6 Working Environment	Q7 Feelings of Fatigue	Q8 Work-Life Balance	Q9 Career	QOL VAS
Q1 workload	1	0.6945	0.3386	0.5710	0.6163	0.5045	0.4931	0.5117	0.3510	−0.5751
Q2 working time		1	0.3050	0.6224	0.6029	0.4891	0.4883	0.5125	0.4852	−0.5312
Q3 collaboration			1	0.3727	0.3422	0.3358	0.2955	0.2865	0.162	−0.2500
Q4 clinical practice				1	0.6209	0.5565	0.4876	0.4708	0.4223	−0.4688
Q5 working conditions					1	0.8324	0.6152	0.6164	0.5834	−0.6705
Q6 working environment						1	0.5817	0.6005	0.5651	−0.7003
Q7 feelings of fatigue							1	0.6105	0.4850	−0.6552
Q8 work-life balance								1	0.5354	−0.7973
Q9 career									1	−0.6150
QOL VAS										1

**Table 2:** Factor analysis results (single-factor solution).

Rotated Factor Loadings; One-Factor Model	
Parameters	Factor 1
Q5 working conditions	0.9063
Q6 working environment	0.8457
Q8 work-life balance	0.7183
Q4 clinical practice	0.7061
Q7 feelings of fatigue	0.7055
Q2 working time	0.7041
Q1 workload	0.6971
Q9 career	0.6464
Q3 collaboration	0.4032

**Table 3:** Factor analysis results (two-factor solution).

Rotated Factor Loadings: Two-Factor Model		
Parameters	Factor A	Factor B
Q6 working environment	0.8813	0.2753
Q5 working conditions	0.7986	0.4489
Q8 work-life balance	0.5546	0.4373
Q7 feelings of fatigue	0.5458	0.4238
Q9 career	0.5425	0.3426
Q2 working time	0.3000	0.8144
Q1 workload	0.3474	0.7211
Q4 clinical practice	0.4412	0.5931
Q3 collaboration	0.2722	0.3065

**Table 4:** Factor analysis (standardized scores).

Standardized Scores			
	One Factor	Two Factors	
Parameters	Factor 1	Factor A	Factor B
Q1 workload	0.0968	−0.1126	0.3269
Q2 working time	0.0997	−0.241	0.5856
Q3 collaboration	0.0344	0.0016	0.0409
Q4 clinical practice	0.1006	−0.0223	0.1692
Q5 working conditions	0.3623	0.3828	−0.0083
Q6 working environment	0.2122	0.6515	−0.3451
Q7 feelings of fatigue	0.1003	0.0519	0.0485
Q8 work-life balance	0.106	0.0533	0.0544
Q9 career	0.0793	0.0628	0.0128

**Table 5:** Factor analysis (standardized scores).

	Work-Related QOL VAS	Simple Sum	Standardized Scores		
			One-Factor Model	Two-Factor Model: Factor A	Two-Factor Model: Factor B
Work-Related QOL VAS	1	−0.7891	−0.7824		−0.469
Simple Sum		1	0.9792	0.8062	0.71
One-Factor Model			1	0.8854	0.6244
Two-Factor Model: Factor A				1	0.196
Two-Factor Model: Factor B					1

## Data Availability

Raw data were generated by an external survey company. The derived data supporting the findings of this study are available from the corresponding author upon reasonable request.

## Supplementary Materials

The following are available online at https://www.mdpi.com/article/10.3390/jmahp13040057/s1, Figure S1: WQMD-9 (original Japanese version). Definition: This figure is included as an appendix to show the original Japanese text version of WQMD-9 questionnaire. English translation provided for informational purposes only in the other reference [7] as Figure 1.

## Abbreviations

The following abbreviations are used in this manuscript:

COSMIN	Consensus-based Standards for the selection of health Measurement Instruments
MBI-HSS	Maslach Burnout Inventory–Human Services Survey
MDs	Medical Doctors
PSS-10	Perceived Stress Scale
QOL	Quality of Life
SF-12	Short-Form Health Survey
VAS	Visual Analogue Scale
WCW-JSS	Warr–Cook–Wall Job Satisfaction Scale
WHO-5	World Health Organization Five Well-Being Index
WLB	Work–Life Balance
WQMD-9	Medical Doctors’ Work-Related Quality of Life Questionnaire
