# Supplementary material for: Criterion-Related Validity and Reliability of a Measurement Tool for Medical Doctors’ Work-Related Quality of Life in Japan
Source: J Mark Access Health Policy. 2025 Nov 14;13(4):57. doi: 10.3390/jmahp13040057 (PMC12641679; doi:10.3390/jmahp13040057)
Supplement: Supplementary file 1 [file jmahp-13-00057-s001.zip › jmahp-3623581-supplementary.pdf]

Journal of
*Market Access
& Health Policy*

MDPI

**医師の仕事関連のQOL調査票**	
	直近1か月のことを想像して回答してください。
**Q1**	**あなたの「仕事量」についてもっともあてはまるものを選んでください。**
	□ 仕事量には問題はない
	□ 仕事量には少し問題がある
	□ 仕事量にはある程度問題がある
	□ 仕事量にはかなり問題がある
	□ 仕事量には極めて問題がある
**Q2**	あなたが「本来の仕事に費やせる時間」についてもっともあてはまるものを選んでく
	□ 本来の仕事に費やせる時間には問題はない
	□ 本来の仕事に費やせる時間には少し問題がある
	□ 本来の仕事に費やせる時間にはある程度問題がある
	□ 本来の仕事に費やせる時間にはかなり問題がある
	□ 本来の仕事に費やせる時間には極めて問題がある
**Q3**	あなたの「同僚（周り）からの協力」についてもっともあてはまるものを選んでくだ
	□ 同僚（周り）から協力を得られている
	□ 同僚（周り）からやや協力を得られている
	□ 同僚（周り）から協力を得られているかどうか、どちらとも言えない
	□ 同僚（周り）からあまり協力を得られていない
	□ 同僚（周り）から協力を得られていない
**Q4**	あなたの「診療」についてもっともあてはまるものを選んでください。
	□ 診療に問題はない
	□ 診療にあまり問題はない
	□ 診療に問題がるかどうか、どちらとも言えない
	□ 診療に少し問題がある
	□ 診療に問題がある
**Q5**	あなたの「労働条件」についてもっともあてはまるものを選んでください。
	□ 労働条件に満足している
	□ 労働条件にやや満足している
	□ 労働条件に満足しているかどうか、どちらとも言えない
	□ 労働条件にあまり満足していない
	□ 労働条件に満足していない
**Q6**	あなたの「労働環境」についてもっともあてはまるものを選んでください。
	□ 労働環境に満足している
	□ 労働環境にやや満足している
	□ 労働環境に満足しているかどうか、どちらとも言えない
	□ 労働環境にあまり満足していない
	□ 労働環境に満足していない
**Q7**	あなたの「疲労感」についてもっともあてはまるものを選んでください。
	□ 疲労感はない
	□ 少し疲労感がある
	□ ある程度疲労感がある
	□ かなり疲労感がある
	□ 極めて疲労感がある
**Q8**	あなたの「ワークライフバランス」についてもっともあてはまるものを選んでくださ
	□ ワークライフバランスに満足している
	□ ワークライフバランスにやや満足している
	□ ワークライフバランスに満足しているかどうか、どちらとも言えない
	□ ワークライフバランスにあまり満足していない
	□ ワークライフバランスに満足していない
**Q9**	あなたの「キャリア」についてもっともあてはまるものを選んでください。
	□ 今のキャリアに満足している
	□ 今のキャリアにやや満足している
	□ 今のキャリアに満足しているかどうか、どちらとも言えない
	□ 今のキャリアにあまり満足していない
	□ 今のキャリアに満足していない

Figure S1. WQMD-9 (original Japanese version). Definition: This figure is included as an appendix to show the original Japanese text version of WQMD-9 questionnaire. English translation provided for informational purposes only in the other reference [7] as Figure 1.
